# Epidemiology of COVID-19 and Predictors of Recovery in the Republic of Korea

**DOI:** 10.1155/2020/7291698

**Published:** 2020-07-30

**Authors:** Ashis Kumar Das, Saji Saraswathy Gopalan

**Affiliations:** ^1^Research Group, The World Bank, Washington DC, USA; ^2^Human Development Department, The World Bank, Washington DC, USA

## Abstract

**Background:**

The recent COVID-19 pandemic has emerged as a threat to global health. Though current evidence on the epidemiology of the disease is emerging, very little is known about the predictors of recovery.

**Objectives:**

To describe the epidemiology of confirmed COVID-19 patients in the Republic of Korea and identify predictors of recovery.

**Materials and Methods:**

Using publicly available data for confirmed COVID-19 cases from the Korea Centers for Disease Control and Prevention from January 20, 2020, to April 30, 2020, we undertook descriptive analyses of cases stratified by sex, age group, place of exposure, date of confirmation, and province. Correlation was tested among all predictors (sex, age group, place of exposure, and province) with Pearson's correlation coefficient. Associations between recovery from COVID-19 and predictors were estimated using a multivariable logistic regression model.

**Results:**

Majority of the confirmed cases were females (56%), 20-29 age group (24.3%), and primarily from three provinces—Gyeongsangbuk-do (36.9%), Gyeonggi-do (20.5%), and Seoul (17.1%). The case fatality ratio was 2.1%, and 41.6% cases recovered. Older patients, patients from provinces such as Daegu, Gyeonggi-do, Gyeongsangbuk-do, Jeju-do, Jeollabuk-do, and Jeollanam-do, and those contracting the disease from healthcare settings had lower recovery.

**Conclusions:**

Our study adds to the very limited evidence base on potential predictors of recovery among confirmed COVID-19 cases. We call additional research to explore the predictors of recovery and support development of policies to protect the vulnerable patient groups.

## 1. Introduction

For the first time, a novel coronavirus disease 2019 (COVID-19) originating from Wuhan in China was reported to the World Health Organization in December of 2019 [1]. This novel coronavirus has taken the form of a major pandemic and has affected almost all major nations in the world. There have been more than 3.6 million confirmed cases and about 252,000 deaths as of May 05, 2020 [2]. The very first COVID-19 case was diagnosed in the Republic of Korea (South Korea) on January 20, 2020 [3]. During the first two months of this global epidemic, South Korea had the second highest cases globally following China. According to the Korea Centers for Disease Control and Prevention (KCDC), there have been 10,804 confirmed cases and 254 deaths due to COVID-19 as of May 05, 2020 [4].

We present the epidemiology of COVID-19 in the Republic of Korea using data from the Korea Centers for Disease Control and Prevention and identify the predictors of recovery from the disease.

## 2. Materials and Methods

### 2.1. Data Source

The data were obtained from the Korea Centers for Disease Control and Prevention's publicly shared sources. The dataset contains information about 3,388 confirmed COVID-19 cases in the Republic of Korea from January 20, 2020, through April 30, 2020. After excluding cases with missing values, 3,299 cases were included in the analysis.

### 2.2. Variables

A confirmed case was defined as a person with laboratory-confirmed positive test. The data contained the following patient details: age (in groups), sex, province, date of diagnosis, mode of exposure, and outcome. There were 11 age groups: below10 years, 10-19 years, 20-29 years, 30-39 years, 40-49 years, 50-59 years, 60-69 years, 70-79 years, 80-89 years, 90-99 years, and above 100 years. We combined the last two age groups to create 90 years and above and thus recategorized age to 10 groups. All seventeen provinces of the Republic of Korea were represented: Busan, Chungcheongbuk-do, Chungcheongnam-do, Daegu, Daejeon, Gangwon-do, Gwangju, Gyeonggi-do, Gyeongsangbuk-do, Gyeongsangnam-do, Incheon, Jeju-do, Jeollabuk-do, Jeollanam-do, Sejong, Seoul, and Ulsan. We categorized the dates of diagnosis by weeks, and they were as follows: 20-26 Jan 2020, 27 Jan-02 Feb 2020, 03-09 Feb 2020, 10-16 Feb 2020, 17-23 Feb 2020, 24 Feb-01 Mar 2020, 02-08 Mar 2020, 09-15 Mar 2020, 16-22 Mar 2020, 23-29 Mar 2020, 30 Mar-05 Apr 2020, 06-12 Apr 2020, 13-19 Apr 2020, 20-26 Apr 2020, and 27-30 Apr 2020. Patients were exposed to potential COVID-19 sources in multiple settings. The settings were nursing home, hospital, religious gathering, call center, community center, shelter and apartment, gym facility, overseas inflow, contact with patients, and others. There were three outcomes: death, recovery, and isolation. The confirmed patients after spending some days in isolation were retested. They were considered recovered only after receiving a negative COVID-19 test.

### 2.3. Statistical Methods

We undertook descriptive analyses for the patient characteristics and presented the results stratified by subgroups for each characteristic. Correlation was tested among all patient characteristics with Pearson's correlation coefficient. Associations between recovery from COVID-19 and predictors (age group, sex, province, and exposure) were estimated using a multivariable logistic regression model. We considered associations statistically significant if the *p* value was below 0.05. The statistical analyses were performed using Python programming language Version 3.7 (Python Software Foundation, Wilmington, DE, USA) and Stata Version 15 (StataCorp LLC, College Station, TX).

## 3. Results

### 3.1. Pattern of the Epidemic

**Figure 1:**
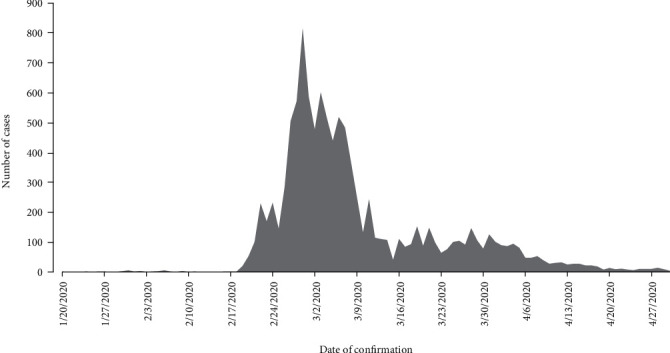
Daily new confirmed COVID-19 cases in the Republic of Korea between January 20, 2020, and April 30, 2020.

As shown in Figure 1, the first case of COVID-19 was confirmed on January 20, 2020. There were a few daily cases of new infections for about a month. After a month, the curve suddenly rose starting February 19, 2020, to reach the peak around end of February and early March. It reached its peak on the 29^th^ of February with 813 confirmed cases. Though the curve descended after this date, still there were on an average 200 daily new confirmed cases until March 11, 2020. The curve continued its downward trend, however, adding at least 100 new daily cases through April 05, 2020. Towards the end of April, daily new confirmed cases were below 10.

### 3.2. Patient Profile

Table 1 shows the profile of the patients. Out of 3,299 confirmed patients, slightly more than half were females (56%). While there were cases from all age groups, the maximum patients were 20-29 years (24.3%), followed by 50-59 years (18.1%), 40-49 years (13.8%), 30-39 years (13.3%), and 60-69 years (12.2%). Three provinces—Gyeongsangbuk-do (36.9%), Gyeonggi-do (20.5%), and Seoul (17.1%)—together accounted for the maximum patients. With respect to the exposure, it was unknown for the most (44%) followed by direct contact with patients (29%), from overseas (16.8%), and religious gathering (4.9%). According to this available data source, 85% percent of the patients were confirmed of their diagnosis between 24 February and 05 April of 2020. There were 61 deaths accounting for 2.1 percent (case fatality rate) of the patients. More than half were isolated (56.3%), and 41.6% recovered.

### 3.3. Predictors of Recovery

**Figure 2:**
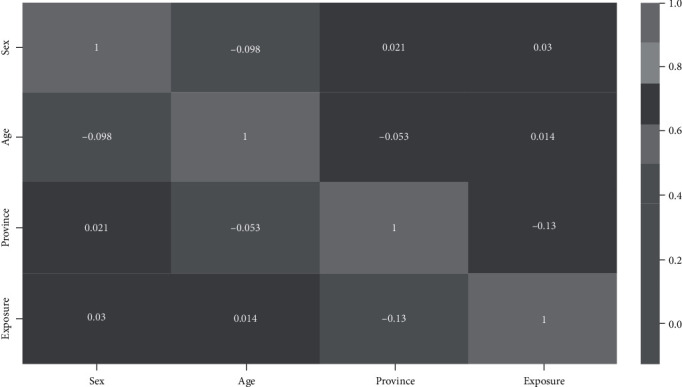
Correlation among predictors. Cells show Pearson's correlation coefficient.

As shown in Figure 2, there were no correlations between the predictors. Both males and females had similar recovery rates, and their difference was not statistically significant (Table 2). Compared to younger age groups, older patients had lower recovery: 70-79 years (adjusted odds ratio 0.31; *p* value 0.01), 80-89 years (aOR 0.22; *p* value 0.001), and 90 years and above (aOR 0.13; *p* value < 0.001). Provinces such as Daegu, Gyeonggi-do, Gyeongsangbuk-do, Jeju-do, Jeollabuk-do, and Jeollanam-do had statistically significant lower recovery rates than Busan. When compared with exposure from nursing homes, patients who were exposed to COVID-19 infection from religious gatherings, community dwellings, and others had higher recovery rates.

## 4. Discussion

Due to multipronged approaches (proactive surveillance, higher testing, isolation, quarantine, use of technology, masks, and social distancing campaigns) by the government, incidence of new cases came down sharply in South Korea by mid-March and further to less than 10 new cases by mid-April [5].

Our study shows that females constituted the majority of confirmed cases, whereas males accounted for most of the confirmed cases in China and Italy [6–9]. Around a fourth of the cases were from the 20-29 years age group unlike in most other countries where the infected were older [6, 7, 10]. As already pointed out by researchers from South Korea, the possible reason for higher representation of younger population in our sample could also be specific exposure to cluster of cases through participation in religious activities or workplaces [5, 11]. As shown in a study undertaken in Europe, population density might have played a role for the number of higher cases in certain provinces [12]. The case fatality rate was much lower (2.1%) compared to other countries such as Italy (13.3%) and China (4%). Similar to findings from several other countries, we found the elderly to be more vulnerable with lower probabilities of recovery [6, 8, 13]. It is quite possible that the presence of preexisting medical conditions in the elderly predispose them to delayed recovery. We also found that cases contracting the infection in nonhealthcare setting had higher recovery. While there is no such evidence currently, there could be a possibility that the exposure outside nonhealthcare setting might have involved relatively younger and healthier cases. Considering our study findings, we suggest additional measures to protect the vulnerable cases who are less likely to recover from the infection. Thus, the elderly and cases contracting infection from healthcare settings should be given special attention.

Our study has two potential limitations. First, we used publicly available data of only a third of confirmed cases in the country. Thus, we are unable to ascertain the representativeness of the data for all confirmed cases in South Korea. So, the findings will have to be interpreted with caution. Second, the data lacks information of patients' symptoms and clinical features. Inclusion of these potential predictors would have enhanced the relevance of this study further. Despite these limitations, our study adds to the very limited evidence base on potential predictors of recovery among confirmed COVID-19 cases [14]. However, we believe the evidence base can be strengthened with further relevant research as authorities make more data publicly available or through primary hospital-based studies.

## 5. Conclusions

The COVID-19 pandemic has emerged as a great threat to global health challenging health systems across the world to efficiently deal with this situation. Emerging evidence on vulnerability to COVID-19 and predictors of recovery will inform providers and policy makers to effectively triage and prioritize limited resources. Therefore, we call for additional research to explore the predictors of recovery and support development of policies to protect the vulnerable patient groups.

## Figures and Tables

**Table 1:** Sample characteristics (*N* = 3,299).

Variable	Number	Proportion (%)
Sex		
Female	1,848	56.0
Male	1,451	44.0
Age group (years)		
Below 10	53	1.6
10-19	149	4.5
20-29	801	24.3
30-39	438	13.3
40-49	454	13.8
50-59	597	18.1
60-69	401	12.2
70-79	204	6.2
80-89	156	4.7
90 and above	46	1.4
Province		
Busan	134	4.1
Chungcheongbuk-do	44	1.3
Chungcheongnam-do	143	4.3
Daegu	63	1.9
Daejeon	40	1.2
Gangwon-do	49	1.5
Gwangju	30	0.9
Gyeonggi-do	677	20.5
Gyeongsangbuk-do	1,218	36.9
Gyeongsangnam-do	112	3.4
Incheon	92	2.8
Jeju-do	13	0.4
Jeollabuk-do	17	0.5
Jeollanam-do	15	0.5
Sejong	46	1.4
Seoul	563	17.1
Ulsan	43	1.3
Exposure		
Nursing home	46	1.4
Hospital	37	1.1
Religious gathering	160	4.9
Call center	112	3.4
Community center, shelter, and apartment	50	1.5
Gym facility	34	1.0
Overseas inflow	553	16.8
Contact with patients	957	29.0
Others	1,350	40.9
Date of confirmed diagnosis		
20-26 Jan 2020	3	0.1
27 Jan-02 Feb 2020	12	0.4
03-09 Feb 2020	12	0.4
10-16 Feb 2020	3	0.1
17-23 Feb 2020	258	7.8
24 Feb-01 Mar 2020	750	22.7
02-08 Mar 2020	651	19.7
09-15 Mar 2020	356	10.8
16-22 Mar 2020	348	10.6
23-29 Mar 2020	347	10.5
30 Mar-05 Apr 2020	349	10.6
06-12 Apr 2020	102	3.1
13-19 Apr 2020	71	2.2
20-26 Apr 2020	30	0.9
27-30 Apr 2020	7	0.2
Outcome		
Died	69	2.1
Recovered	1,372	41.6
Isolated	1,858	56.3

**Table 2:** Predictors of recovery.

Variable	Recovery (%)	Odds ratio	95% confidence interval		*p* value
Sex					
Female	56.6	Reference			
Male	56.0	0.90	0.73-1.10		0.312
Age group (years)					
Below 10	60.4	Reference			
10-19	58.4	1.33	0.55-3.25		0.527
20-29	46.8	1.51	0.68-3.36		0.314
30-39	50.7	1.77	0.78-4.04		0.173
40-49	38.8	1.13	0.50-2.56		0.771
50-59	39.5	1.02	0.45-2.31		0.955
60-69	44.4	0.81	0.35-1.84		0.611
70-79	38.7	0.31	0.13-0.76		0.01
80-89	32.7	0.22	0.09-0.54		0.001
90 and above	32.6	0.13	0.04-0.37		<0.001
Province					
Busan	82.8	Reference			
Chungcheongbuk-do	88.6	1.02	0.35-2.99		0.971
Chungcheongnam-do	88.8	1.38	0.61-3.12		0.432
Daegu	6.4	0.00	0.00-0.01		<0.001
Daejeon	85.0	1.38	0.43-4.44		0.585
Gangwon-do	59.2	0.30	0.12-0.78		0.013
Gwangju	70.0	0.67	0.25-1.79		0.424
Gyeonggi-do	9.0	0.01	0.01-0.02		<0.001
Gyeongsangbuk-do	63.5	0.10	0.05-0.19		<0.001
Gyeongsangnam-do	85.7	0.93	0.40-2.15		0.866
Incheon	59.8	0.55	0.26-1.18		0.125
Jeju-do	53.9	0.29	0.08-1.00		0.05
Jeollabuk-do	23.5	0.03	0.01-0.11		<0.001
Jeollanam-do	20.0	0.03	0.01-0.13		<0.001
Sejong	87.0	1.20	0.25-5.86		0.821
Seoul	74.1	0.70	0.38-1.29		0.257
Ulsan	86.1	1.24	0.32-4.83		0.76
Exposure					
Nursing home	17.4	Reference			
Hospital	43.2	1.14	0.34-3.85		0.833
Religious gathering	80.6	6.29	1.80-21.94		0.004
Call center	84.8	2.68	0.77-9.31		0.122
Community center, shelter, and apartment	82.0	13.34	3.06-58.05		0.001
Gym facility	94.1	6.05	0.63-57.79		0.118
Overseas inflow	40.9	2.71	0.90-8.13		0.075
Contact with patients	41.5	2.80	0.98-8.03		0.055
Others	67.7	7.14	2.58-19.75		<0.001

## Data Availability

The data used to support the findings of this study are available publicly through the Korea Centers for Disease Control and Prevention.
